# Mechanism of action, resistance, interaction, pharmacokinetics, pharmacodynamics, and safety of fostemsavir

**DOI:** 10.1186/s12879-024-09122-5

**Published:** 2024-02-23

**Authors:** Mohsen Heidary, Saeedeh Shariati, Shima Nourigheimasi, Mona Khorami, Melika Moradi, Moloudsadat Motahar, Parisa Bahrami, Sousan Akrami, Vahab Hassan Kaviar

**Affiliations:** 1 Department of Laboratory Sciences, School of Paramedical Sciences, Sabzevar University of Medical Sciences, Sabzevar, Iran; 2 Cellular and Molecular Research Center, Sabzevar University of Medical Sciences, Sabzevar, Iran; 3 Toxicology Research Center, Medical Basic Sciences Research Institute, Ahvaz Jundishapur University of Medical Sciences, Ahvaz, Iran; 4 Student Research Committee, Ahvaz Jundishapur University of Medical Sciences, Ahvaz, Iran; 5 Faculty of Medicine, Arak University of Medical Science, Arak, Iran; 6 Department of Obstetrics and Gynecology, School of Medicine, Ilam University of Medical Sciences, Ilam, Iran; 7 Department of Microbiology, School of Medicine, Ahvaz Jundishapur University of Medical Sciences, Ahvaz, Iran; 8 Students’ Scientific Research Center (SSRC), Tehran University of Medical Sciences, Tehran, Iran; 9 Department of Microbiology, School of Medicine, Tehran University of Medical Sciences, Tehran, Iran; 10 Clinical Microbiology Research Center, Ilam University of Medical Sciences, Ilam, Iran

**Keywords:** Fostemsavir, Resistance, Human immunodeficiency virus, Antiretroviral therapy, HIV, AIDS

## Abstract

The Food and Drug Administration (FDA) has licensed many antiretroviral medications to treat human immunodeficiency virus type 1 (HIV-1), however, treatment options for people with multi-drug resistant HIV remain limited. Medication resistance, undesirable effects, prior tolerance, and previous interlacement incapacity to deliver new drug classes all lead to the requirement for new medication classes and drug combination therapy. Fostemsavir (FTR) is a new CD-4 attachment inhibitor medicine that was recently authorized by the United States FDA to treat HIV-1. In individuals with multidrug-resistant (MDR) HIV-1, FTR is well tolerated and virologically active. According to recent investigations, drug combination therapy can positively affect MDR-HIV. The mechanism of action, resistance, interaction, pharmacokinetics, pharmacodynamics, and safety of FTR has been highlighted in this review.

## Introduction

HIV-1, a retrovirus, can induce acquired immunodeficiency syndrome if not treated. According to data from the World Health Organization (WHO), an estimated 39 million individuals worldwide are living with HIV-1 [1–4]. Because HIV-1 has significant genetic diversity, monotherapy with the authorized therapies for this virus frequently leads to the selection of resistant variants. However, combined antiretroviral treatment (cART) combining various classes of HIV-1 medications has been confirmed to be extremely successful in HIV-1-positive individuals, making the infection a long-term chronic condition [5, 6].

However, the emergence of drug-resistant and multidrug-resistant strains remains a key factor in the failure of cART, leading to higher odds of HIV disease progression and mortality [7]. HIV medications are classified into six types based on whether they target viral proteins or the virus’s attachment to host cells. These categories include nucleoside/nucleotide reverse transcriptase inhibitors (NRTIs), protease inhibitors (PIs), non-nucleoside reverse transcriptase inhibitors (NNRTIs), entrance inhibitors, integrase strand transfer inhibitors (INSTIs), and capsid inhibitors. Entry inhibitors are further classified as pre-attachment inhibitors, post-attachment inhibitors, CCR5 antagonists, and fusion inhibitors [8].

New mutations cause limitations of cART drug regimen. Other limitations of this treatment regimen include the high cost of treatment and drug-drug interactions [9, 10].. According to the World Health Organization’s most current HIV Drug Resistance Report, the prevalence of acquired and transmitted HIV drug resistance has increased significantly and rapidly among people who have not yet received ART. This growth presents a significant barrier to attaining the aim of eliminating the HIV-1 pandemic as a public health issue by 2030. According to the findings, 10% of HIV-positive persons who begin HIV therapy are already resistant to non-nucleoside reverse transcriptase inhibitors (NNRTI) [11].

Pre-attachment inhibitors, such as FTR, hinder the virus’s first attachment to host cells by inhibiting interactions between gp120 and CD4 [12]. Post-attachment inhibitors, such as ibalizumab, promote alterations in the CD4-gp120 complex, blocking fusion between the virus and the cell [13, 14]. CCR5 antagonists, such as maraviroc and leronlimab, prevent viral docking by binding to the CCR5 co-receptor [15]. Fusion inhibitors, such as the synthetic peptide enfuvirtide (T-20; ENF), are a kind of ARV that prevents the virus from entering host cells [16].

FTR, originally BMS-663,068/GSK-3,684,934, is an FDA-approved medication marketed under the brand name Rukobia [17].

FTR attaches to the viral envelope glycoprotein 120 (gp120), inhibiting the conformational shift required for the virus to enter host T cells and other immune cells [18, 19]. During its development, it was revealed that FTR is converted into a more permeable derivative termed TMR by the action of alkaline phosphatase near the surface of the gut epithelium [18, 20, 21].

HIV-MDR has a limited response to most existing HIV medicines, making it difficult to develop effective treatment regimens to reduce viral replication in these individuals. HIV-MDR infection has been associated with increased risks of clinical deterioration and fatality [22, 23]. Individuals who have previously used ARV medications have a threefold increased probability of acquiring resistance to the NNRTI drug class [24, 25]. The BRIGHT trial’s findings did not consistently demonstrate a link between virologic failure (the inability to reduce viral replication) and particular gp120 mutations. Furthermore, there have been no instances of FTR developing cross-resistance to other ARV medications. FTR is considered an essential therapy option for individuals with HIV who are resistant to other drugs, perhaps saving lives [26–29].

## Antiviral features

### Structure of drug

**Fig. 1:**
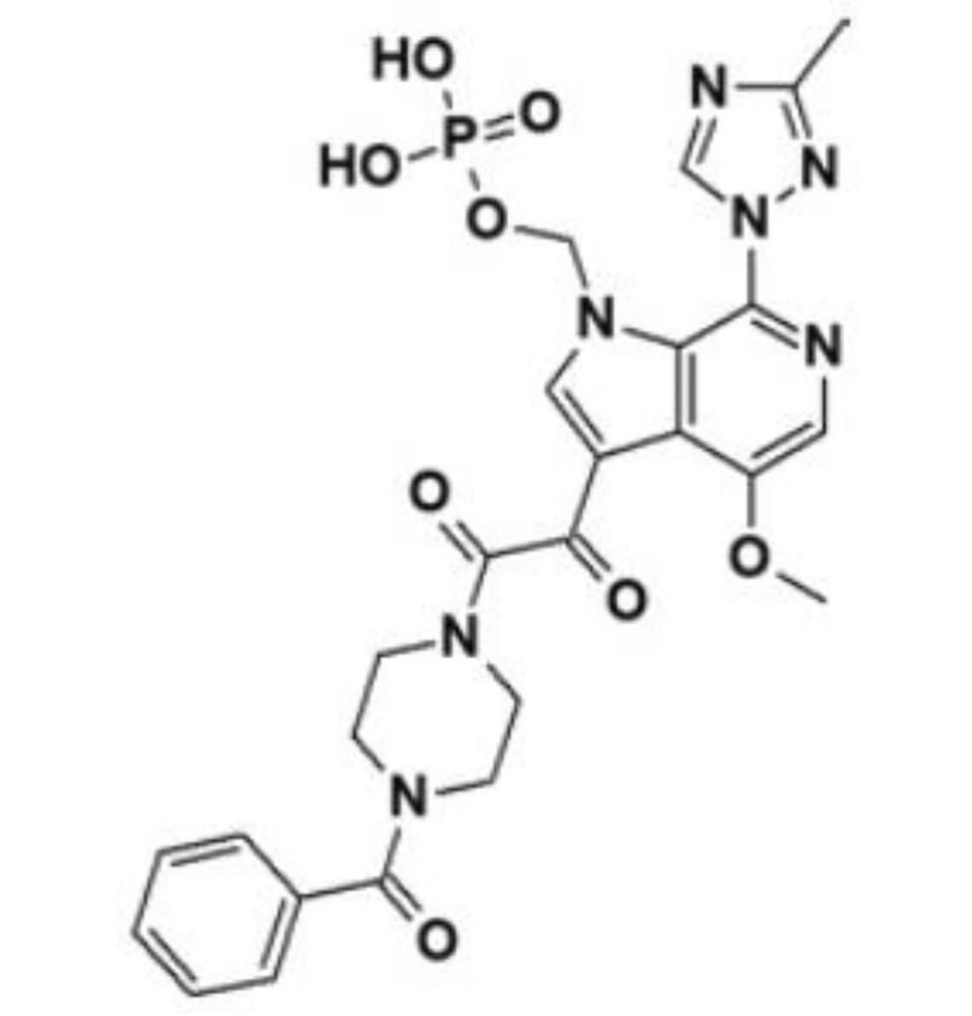
Structure of fostemsavir (The figure was adopted and reproduced from Lai et al. with permission from the publisher) [5]

FTR tromethamine is chemically named as (3-((4-benzoyl-1-piperazinyl) (oxo)acetyl)-4-methoxy-7-(3-methyl-1 H-1,2,4-triazol-1-yl)-1 H-pyrrolo[2,3-c] pyridin-1-yl) methyl dihydrogen phosphate or 2-amino-2-(hydroxymethyl)-1,3-propanediol and corresponds to the molecular formula C25H26N7O8P.C4H11NO3. FTR is in the form of salt and has a relative molecular mass of 704.62 g/mol. Its chemical structure is depicted in Fig. 1.

Following the oral administration, FTR, before absorption, undergoes a fast and widespread metabolism by alkaline phosphatase on the luminal surface of the small intestine brush border membranes and releases TMR, most probably through an unstable N-hydroxymethyl intermediate. Temsavir is absorbed rapidly owing to its great lipophilicity and favorable membrane permeability [30].

### Mechanism of action of Fostemsavir

**Fig. 2:**
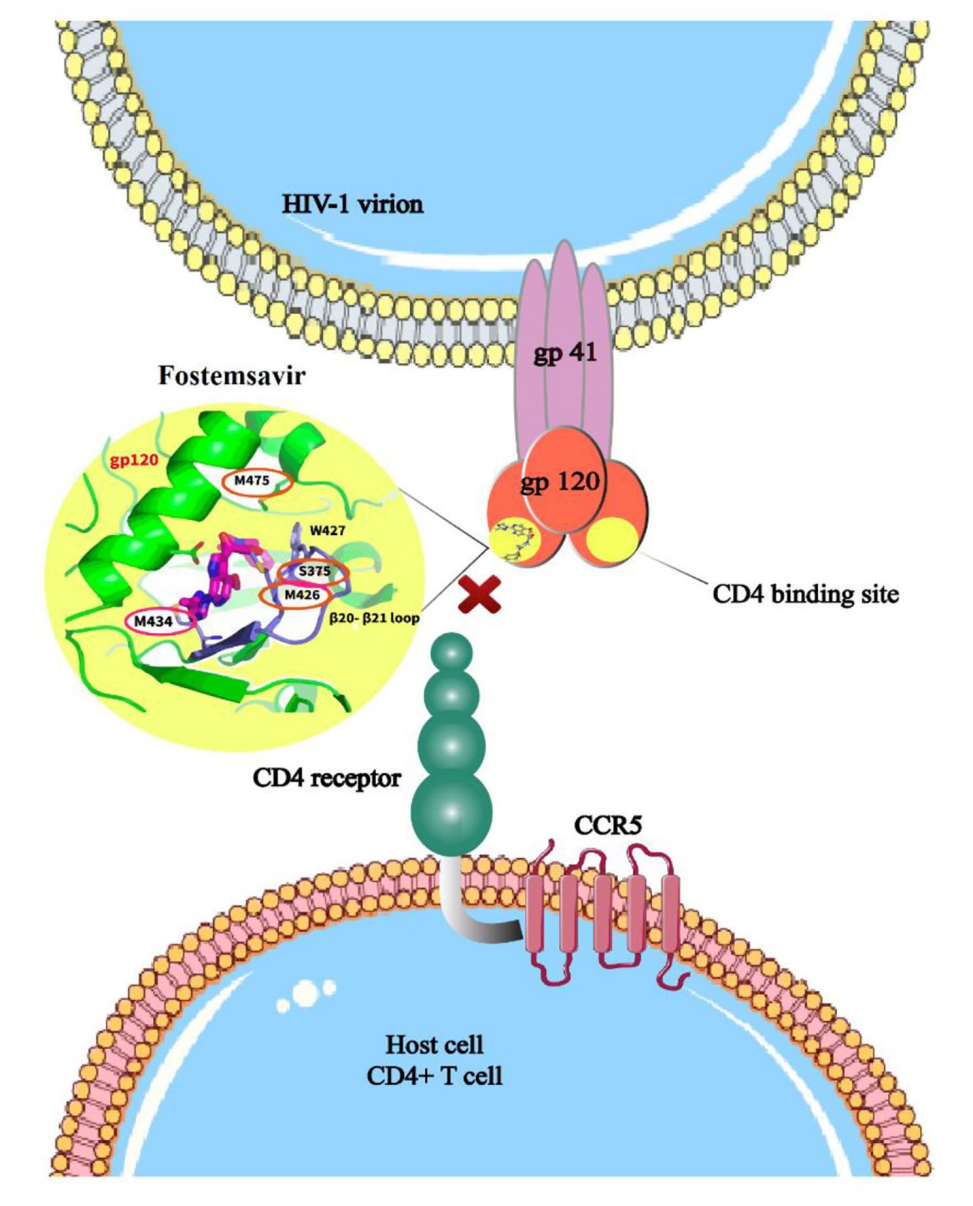
Mechanism of action of fostemsavir. Fostemsavir inhibits the viral interaction with host CD4 receptors by direct binding to the gp120 subunit, thereby inhibiting the initial attachment needed for the replication of virus. It also prevents other gp120-dependent post-attachment steps needed for the entry of virus. It is predicted that the inhibitor of fostemsavir binds adjacent to the CD4 binding loop and β20-β21 hairpin. Indeed, the binding of this drug inhibits the interaction with CD4 and stabilizes Env in a prefusion “closed” state 1 conformation, which is preferably targeted bNAbs

FTR is an attachment inhibitor (Fig. 2). Various data on TMR-related inhibitors in complexes with HIV-gp120 have been suggested for providing a structural basis for the inhibition [19, 31]. In these models, it is predicted that the inhibitor of TMR binds adjacent to the CD4 binding loop and β20-β21 hairpin. Indeed, the binding of this drug inhibits the interaction with CD4 and stabilizes Env in a prefusion “closed” state, which is preferably targeted by broadly neutralizing antibodies (bNAbs). At physiological concentrations, a change in Env glycosylation and cleavage by TMR significantly alters the total antigenicity of Env, thereby decreasing the bNAbs capacity to identify and remove HIV-1-infected cells by antibody-dependent cellular cytotoxicity (ADCC) [32].

#### Mechanism of action of non-fostemsavir

Enfuvirtide, a synthetic peptide, is also a class of ARVs that inhibits the entry of the virus into host cells. It has several defects that limit its clinical use, including relatively low antiviral activity, requiring high doses, a lower genetic barrier for drug resistance, and a short in vivo half-life [16]. Entry inhibitors with novel mechanisms of action and greater convenience in dosing would be useful. HIV entry is conducted via three steps. In the first step, HIV binds to the CD4 receptor. In the next step, HIV binds to CC chemokine receptor 5 (CCR5) or C-X-C chemokine receptor type 4 (CXCR4), and in the last step, the viral and host cell membranes are fused [33].

### FTR activity against drug-resistant HIV

In a phase I, 8-day evaluation dose-escalation trial, on 48 HIV-1 subtype B treatment-naive subjects, FTR gave an average decrease of HIV RNA of 1.21–1.73 log10 copies/mL with a good safety profile [34]. Also, AI438011 is a phase IIb, randomized trial in which 251 ARV-experienced patients were randomized 1:1:1:1:1 into five arms: FTR (400 mg twice daily, 800 mg twice daily, 600 mg once daily, or 1,200 mg once daily) and ritonavir-boosted atazanavir (ATV/r) 300/100 mg once daily, each with a backbone of raltegravir 400 mg twice daily plus tenofovir disoproxil fumarate 300 mg once daily. Through week 48, on the modified intention-to-treat (mITT) analysis, the proportion of patients with HIV-1 RNA < 50 copies/ml turned into 61–82% inside the FTR groups and 71% for ATV/r arms. Response rates observed in subjects with a baseline viral load of less than 100,000 copies/mL were 74–100% versus 96% (FTR versus ATV/r group) and 60–91% versus 71% for baseline viral loads ≥ 100,000 copies/ml [35]. FTR has an exceptional resistance profile and no in vitro cross-resistance has been observed with other classes of ARVs, including nucleoside reverse transcriptase inhibitors (NRTIs), non-NRTIs (NNRTIs), protease inhibitors (PIs), integrase inhibitors, CCR5 antagonists, and fusion inhibitors [36].

In an international phase 3 trial, FTR was investigated in patients with multidrug-resistant (MDR) HIV-1 infection. The patients (*n* = 371) were divided into two groups. The first (randomized) group (in a 3:1 ratio; *n* = 272) used a minimum of one fully active, approved ARV drug in at least one but no more than two ARV classes adding either FTR or placebo to their failing regimen for eight days. They commenced open-label FTR in combination with optimized background therapy on day one. On day eight, the mean reduction in the HIV-1 RNA level was 0.79 and 0.17 log_10_ copies/ml in the FTR and placebo groups, respectively (*P* < 0.001). At week 48, a virologic response (HIV-1 RNA level, < 40 copies/ml) happened in 54% and 38% of the patients in the randomized and nonrandomized groups, respectively. The mean increase in the CD4^+^ T-cell count was respectively 139 and 64 cells per cubic millimeter. In patients with MDR-HIV-1 infection with limited treatment choices, the HIV-1 RNA level had a considerably larger decrease in those who got FTR than in those who received a placebo within the first eight days. Efficacy remained up to 48 weeks [27]. The virological failure rate of 18% at week 48 in the randomized cohort (RC) was similar to that reported in other studies of patients with MDR HIV infection [37, 38].

In a single-group, phase 3 study involving patients with MDR HIV-1 infection, ibalizumab was assessed. Of the 40 patients in the intention-to-treat population, 83% had a decrease in viral load of 0.5 log_10_ copies per milliliter from baseline. Also, the mean CD4 count increased from 150 cells per microliter at baseline (as measured in 40 patients) to 240 cells per microliter at week 25 (as measured in 27 patients), with a mean increase of 62 cells per microliter. These findings were less than the results of FTR in that the mean increase in the CD4 + T-cell count was 139 cells/mm^3^ and the mean reduction in the HIV-1 RNA level was 0.79 log_10_ copies/ml. To construct an optimized background regimen with at least one fully susceptible ARV agent, 17 patients (43%) required the addition of an investigational ARV drug. They found that ibalizumab combined with an optimized regimen had antiviral and immunologic activity [37].

Additionally, in a phase 3 study using dolutegravir (DTG) 50 mg twice daily in raltegravir-resistant and/or elvitegravir-resistant HIV-1 patients, the mean increase in CD4 + T cells at week 48 was 125 cells/mm^3^ [38]. Ackerman et al. reported a response rate of 24 to 96 weeks in a study with FTR 600 mg twice daily and using optimized background therapy (OBT) in heavily treatment-experienced individuals failing ART with limited treatment options virologic. Viral response was most clearly associated with overall susceptibility to new OBT agents. CD4 + T-cell count increases were significant, and a mean increase of 240 cells/µl [39].

In addition, FTR was tested as a possible component of ART in HIV eradication strategies in both in vivo humanized mice and in vitro peripheral blood mononuclear cells from HIV-infected patients. Finally, FTR showed a reassuring safety profile without significant effects on metabolic parameters [40]. In treatment-experienced individuals with advanced HIV-1 disease and limited treatment options, FTR-based ARV regimens were generally well-tolerated. It showed a distinctive trend of increasing virological and immunological response rates through 96 weeks [28].

About FTR susceptibility, Zuze et al.‘s 2023 study [41], revealed a low prevalence of FTR-associated resistance, establishing a baseline for potential polymorphisms despite limitations in predicting their impact. Despite these limitations, the study underscores FTR as a valuable option for individuals failing current ARV regimens, aligning with the UNAIDS 95-95-95 goals and addressing drug resistance challenges in HIV treatment. In this study prevalence of FTR polymorphisms was comparable in both ART-experienced and ART-naïve persons with virologic failure in a setting with no prior FTR exposure. Rose and colleagues [42] presented clinical evidence supporting the lack of cross-resistance between TMR, and ibalizumab or maraviroc. Their study affirmed that decreased susceptibility to TMR and resistance to ibalizumab or maraviroc were not linked, suggesting the potential utility of FTR in individuals with failure on these entry-targeted agents.

Additionally, Saladini et al., [43] demonstrated that FTR maintained efficacy even in MDR HIV-1 variants, with comparable IC50 values to wild-type viruses. Exposure to entry inhibitors and viral tropism did not affect TMR susceptibility. Li et al., [44] investigated FTR against CD4-independent viruses and HIV-1 envelopes resistant to other entry inhibitors. Although some association with maraviroc resistance was noted, there was no absolute correlation, emphasizing the lack of cross-resistance between FTR and other HIV entry inhibitors, indicating potential sequential or concurrent use with different classes.

## FTR resistance

### Mechanisms of resistance

Changes in the HIV genetic structure cause alterations in the ability of medications to inhibit virus replication. Due to the emergence of drug-resistant virus strains, almost all available ARV medicines are prone to become partially or fully inactive [45]. The main mechanism known for FTR resistance has been ascribed to the mutations of the *env* gene, encoding the gp160 glycoprotein and then cleaving into the viral envelope proteins gp41 and gp120, which allows the virus to attach to target cells through binding to the CD4 receptor [46]. A number of the gp120 residues that interact with Bristol-Myers Squibb compounds influence the susceptibility of drugs when altered or induced drug resistance in vitro [47]. There are reports of FTR drug resistance; certain substitutions can cause the loss of hydrophobic interactions between drug and gp120 or decrease the size of the binding site, which restricts the ability of FTR to access its binding sites [47–50].

### Emergence of genotypic resistance and changes in FTV susceptibility under treatment

There is a continuing need for the development of new classes of ARVs with unique mechanisms of action that are well tolerated and do not cross-resistance to current medications for this group [51]. BRIGHTE (NCT02362503) is a multicenter, two-cohort, phase 3 study that is now being conducted at 108 sites in 22 countries. They enrolled heavily treated adults who were failing ART (HIV-1 RNA 400 copies per mL) into two cohorts: the RC, which received oral FTR or placebo in combination with their failing regimen for 8 days, followed by FTR plus optimized background therapy; or the NRC, in which patients with no other ARV alternatives started on day 1 with oral FTR plus optimized background treatment. Rates of virological suppression (HIV-1 RNA 40 copies per mL) rose from 53% at week 24 to 60% at week 96 in the randomized group. At weeks 24 and 96, the NRC response rate was 37%. The RC’s mean increase in CD4 count from baseline at week 96 was 205 cells per L while the NRC’s was 119 cells per L. FTR-based ARV regimens were generally well tolerated and showed a distinct trend of increasing virological and immunological response rates through 96 weeks in heavily treated individuals with advanced HIV-1 disease and limited treatment options; these findings support FTR as a treatment option for this vulnerable population [28]. In continuation of the previous study, a study conducted by Gartland et al. includes genotypic and phenotypic analysis of HIV-1 samples from 63/272 (23%) RC participants and 49/99 (49%) NRC individuals who satisfied protocol-defined virologic failure (PDVF) criteria through 96 weeks. The incidence of PDVF was as expected in this difficult-to-treat patient population, and it was comparable among RC participants regardless of the presence of predefined gp120 amino acid substitutions that may influence phenotypic susceptibility to TMR (S375H/I/M/N/T, M426L, M434I, M475I) or baseline TMR 50% inhibitory concentration fold change (IC50 FC). The frequency of PDVF was lower in patients who had higher overall susceptibility scores to freshly used ARVs (OSS-new), indicating that OSS-new may be a better predictor of virologic outcomes in people who have had a lot of therapy. Predefined gp120 alterations, most notably M426L or S375N, emerged on therapy in 24/50 (48%) RC and 33/44 (75%) NRC subjects with PDVF, with TMR IC50 FC increasing. In the first optimized background therapy of BRIGHT, PDVF was not consistently related to treatment-emergent genotypic or phenotypic alterations in susceptibility to TMR or ARVs [52].

### Epidemiology of resistance

Resistance to FTR, the same as other medicinal products, appeared early. In study performed by Lataillade et al., the effectiveness, safety, and dose-response of FTR were investigated in 200 HIV-1-infected patients. Through week 48, 66 (33%) out of 200 FTR-treated cases met the criteria for resistance testing. Genotyping was carried out in 179 out of 200 FTR-treated subjects. Of these 179 cases, 75 (42%) had a virus with a substitution at ≥ 1 of the four positions (key positions S375, M426, M434, or M475). Substitutions were related to a < 3 or > 3-fold decrease in the susceptibility to FTR [36]. These outcomes were comparable to the findings in the phase 2a AI438001 trial [43, 53]. The study of the *env* sequence from individuals with alterations in FC-IC_50_ (the 50% maximal inhibitory concentration) of greater than three-fold, who were exposed to FTR in vitro showed eight substitutions (in gp120), comprising S375M/H/T, M426L, M434I, S475I, L116P/Q, A204D, and V506M [53–55]. The first four substitutions were also detected by in vivo experiments [36, 50, 56]. Moreover, the substitutions M426L, M434I, and M475I, explored for the earlier drugs BMS-378,806 and BMS-488,043 as signature mutations, were detected in vitro following treatment with both drugs. M426L and M475I were the substitutions indicated strong resistance of more than 100-fold change to BMS-378,806, but M434I demonstrated minor fold changes. Due to the similarity of the structures of these drugs, their resistance profiles overlap [50].

From the genetic point of view, HIV-1 differences in O, N, and P (HIV-1 non-M) groups are diverse, endemic viruses abundant in Central West Africa and observed occasionally in other regions of the world [44]. The genetic variations of HIV-1 non-M virus in gp120 affirm the concept that they can be highly resistant to FTR. Full genetic susceptibility may happen only in 5% of strains in group O [55]. In vitro and clinical investigations have displayed a genotype relationship with decreased sensitivity to FTR. Resistance related to the CRF01_AE subtype appears to be linked to the natural presence of substitutions S375H and M475I [53].

This resistance of HIV-1/M subtype and the polymorphism of other subtypes highlight the direct impact of genetic diversity on drug effects [55]. Evidence of phenotypical resistance in two HIV-1/O strains has revealed that all HIV-1/O strains are likely resistant to FTR. However, the two strains do not have a full genetic background, possibly influencing drug resistance owing to the high intergroup genetic diversity [57]. In study performed by Kozal et al., the gp120 substituted mutations were found in 20 out of 47 (43%) patients with MDR HIV-1 infection. S375N and M426L were the most common substations. From baseline to virologic failure, the IC_50_ of TMR was enhanced by a factor of 2.3 compared with reference virus [27].

In an earlier study, according to HIV subtype and tropism, *env* mutations were investigated at sites associated with FTR resistance in subjects with a novel HIV-1 diagnosis. The incidence of substitutions in resistant cases was 13.2% (S375T), 6.8% (M426L), 2.9% (M434I), 2.7% (M475I), 1.0% (S375H)/0.8% (M), and 0.31% (L116P) [57]. Based on the Los Alamos National Laboratory HIV Sequence Database for the frequency of polymorphisms at gp160 positions of interest, among all 7560 sequences, have been identified specific polymorphisms that reduce the susceptibility of TMR (S375H/I/M/N/T/Y, M426L/P, M434I/K, and M475I) and have been found in < 10% of isolates of subtypes D, G, A6, BC, F1, CRF07_BC, CRF08_BC, 02 A, CRF06_cpx, F2, 02G, and 02B. Among CRF01_AE subtypes, the substitutions S375H (99.3%) and M475I (76.3%) were predominant, which supports formerly reported low TMR susceptibility of this CRF [58].

The frequency of FTR resistance was evaluated in sequences from 1997subjects. The occurrence of FTR resistance mutations was 0.05% (L116Q), 0.55%/1.35%/17.73% (S375H/M/T; the latter is far less relevant in determining resistance), 7.56% (M426L), 4.21% (M434I), and 1.65% (M475I). Moreover, the prevalence of M426R polymorphism was 16.32%. A significantly higher prevalence in X4 versus R5 viruses was demonstrated only for S375M (0.69% versus 3.93%) and S375T (16.60% versus 22.11%), with *P* = 0.009 and *P* = 0.030, respectively [26]. In study conducted by Charpentier et al., the frequency of substitutions in gp120 was evaluated in 85 patients infected with HIV. The M426L and M434I substitutions were detected in viruses from 10 (11.8%) and 11 (12.9%) patients [59].

In another study, the prevalence of mutations (L116P, A204D, S375M/H, M426L, M434I, M475I, and V506M) related to decreased sensitivity to FTR was surveyed in 111 gp120 sequences from groups O (*n* = 100), N (*n* = 9), and P (*n* = 2). S375M, M426L, and M434I were three substitutions detected in all HIV-1 group N sequences. However, 1% of HIV-1 group O sequences had the S375H + M426L pattern, while 10% had S375H + M434I pattern [48]. In a 2020 survey, 23 out of 24 gp120 sequences from patients with MDR strains, mutations associated with TMR resistance were observed in two M426L and one S375N case(s) [43].

In a survey conducted in France, sequences from 109 attachment-inhibitor-naive patients infected with HIV-1 subtype B were analyzed to detect the presence of in vivo FTR-associated resistance mutations and to determine tropism. The M426L substitution associated with the decreased efficacy of the FTR was found in 7.3%. Also, according to virus tropism (R5 or X4), no difference was identified in mutation distribution. The results revealed that the attachment inhibitor FTR is appropriate for the majority of patients infected with HIV-1 subtype B [55].

## Interaction of FTR with ARVs

Evidence has indicated few significant interactions between FTR and other ARVs or medications frequently used for HIV-associated comorbidities. FTR is often metabolized by a hydrolysis pathway mediated by esterase. Also, it is partly metabolized through an oxidative pathway mediated by CYP3A4. Researchers and collaborators at ViiV Healthcare (USA) compiled data from 11 studies on drug-drug interaction to report the potential use of FTR with other ARVs and with drugs commonly used by HIV-infected people. Strong CYP3A inducers (e.g., rifampin) and anti-HCV drug (e.g., elbasvir/grazoprevir) are contraindicated with FTR. As the metabolization of TMR is performed by CYP3A4, strong CYP3A inducers considerably mitigate TMR plasma concentrations and may cause impairment in virologic response to FTR. TMR prevents the transporter OATP1B1/3, which may result in higher grazoprevir concentrations, therefore increasing liver enzymes [60].

The Data Documentation Initiative from 13 investigations was collected to report the effect of 17 drugs or drug combinations on TMR. FTR with CYP3A4, P-glycoprotein (P-gp), and/or breast cancer resistance protein (BCRP) inhibitors raised TMR concentrations; however, they do not cause clinical concern at therapeutic dose. TMR may be given with weak/moderate inducers with or without co-administration, such as ritonavir (RTV) or cobicistat (COBI). FTR can be administered with drugs raising stomach pH; famotidine did not influence TMR pharmacokinetics (PK). Temsavir may enhance the concentrations of drugs that are substrates of organic-anion transporting polypeptide 1B1/3 and BCRP. Thus, the majority of statins need dose reduction [61]. Wire et al., showed that co-administration of maraviroc and FTR did not result in clinically relevant changes in TMR. These two drugs may be co-administered without dose adjustment of either ARV agent [62].

High concentrations of grazoprevir may enhance the risk of alanine aminotransferase (ALT) elevations. Thus, administration of FTR with elbasvir/grazoprevir is not recommended. Dose alterations and/or careful titration of dose are recommended for certain statins that are substrates of OATP1B1/3 or BCRP (rosuvastatin, atorvastatin, pitavastatin, simvastatin, and fluvastatin) when co-administered with FTR. Following the co-administration of FTR with tenofovir/alafenamide (TAF), TMR is speculated to raise the plasma concentrations of TAF by blocking OATP1B1/3 and/or BCRP. The recommended TAF dose is 10 mg when co-administered with FTR. Due to drug interaction of COBI and RTV, FTR may be administered combined with strong CYP3A4, BCRP, and/or P-gp inhibitors (such as clarithromycin, itraconazole, posaconazole, and voriconazole) without dose adjustment [63].

One study investigated 99 patients who had no active drugs as treatment options. However, FTR was added to their optimized ARV regimen. Of these 99 patients, 38 cases obtained an HIV viral load of < 40 copies/mL at 48 weeks. Co-administration of FTR with strong cytochrome P450 3 A inducers is contraindicated as the plasma concentrations of TMR considerably decreased. Etravirine, another ARV agent, may lower TMR plasma concentrations. However, after its combination with a ritonavir-boosted protease (strong) inhibitor, the impact on TMR metabolism is insignificant and does not need dose alteration [64].

## Pharmacokinetics and pharmacodynamics

### Pharmacokinetics

Pharmacokinetic analysis of two phase 2 clinical trials, AI438006 and AI438011, confirmed an appropriate pharmacokinetic profile for FTR. The 600 mg dose of FTR twice daily (BID, bis in die = means twice a day), in comparison to the 800 mg BID and the 1200 mg once daily doses, seems to cause a lesser maximum plasma concentration. Moreover, it can readily increase the level of the supratherapeutic window and lessen the risk of QTc (Corrected QT interval) interval prolongation [65].

Temsavir has an average plasma half-life of 3.2–4.5 h (immediate) or 7–14 h (extended) release formulation and is eliminated through hydrolysis and partly through oxidation, which is mediated by hepatic esterase and by cytochrome P450 3A4, respectively. The excretion of primary metabolite happens through urine (44–51%), feces (33%), and bile (5%) [66]. Based on these outcomes, the 600 mg BID dose was recommended for the phase 3 trial. FTR in combination with CYP3A4 inhibitors or CYP3A4 inducers has the potential for drug-drug interactions [67, 68]. There is no report on the pharmacokinetics of FTR in individuals with hepatic failure and renal insufficiency. Also, no report has yet been reported on the impact of eating on the pharmacokinetics of FTR [69]. TMR exposure increased with a single 600 mg oral dosage of FTR, although apparent clearance decreased. It was associated with increased severity of hepatic failure in healthy people who had mild, moderate, or severe hepatic failure. Temsavir was 79.9% protein-bound in individuals with mild, 81.9% in moderate, and 76.5% in severe hepatic failure, as opposed to 81% in subjects with normal renal function [70].

### Pharmacodynamics

FTR indicated an ameliorated inhibitory quotient and a greater obstacle for the selection of resistance more than its precursor, namely BMS-488,043. The antiviral activity of this agent changes with 50% inhibitory concentration (IC_50_), decreased plasma concentration, and stable-state plasma TMR concentrations. In HIV-1 RNA viral load, decreased IC_50_ (< 100 nmol) was initially related to a reduction $\ge$1 log10, but greater baseline IC_50_ (> 100 nmol) mainly had a link to a reduction <1 log10. Among the AE and O.14 subtypes, the prevalence of TMR resistance is mostly associated with the gp120 heterogeneity. In Molina et al.’s investigation, superb activity was observed against the majority of subtypes, except for two subgroups, i.e. AE and B, which had no geographical variations [71]. Another survey explored that among 20 varied subtype envelopes, there is a wide range of variability with a prevalent high susceptibility to TMR (IC_50_ <100 nmol), regardless of CRF01_AE viruses, which exhibited IC_50_ >100 nmol in all five cases. However, the subtype, IC_50_, or existence of at-risk polymorphisms at positions M475, S375, M434, and M426 possibly reduce susceptibility in a condition-dependent way and cannot make a clear prediction about virological response in clinical trials [72]. In some patient, particularly those who was clinically considerable at four times (1200 mg daily), QTc interval prolongation dependent on TMR concentration was found on electrocardiogram (ECG). Hence, during therapy, patients with a history of QTc prolongation, concurrent consumption of QTc prolongation drugs, or heart diseases need to be treated cautiously and should be supervised by ECG [73].

## Safety, efficacy and side effects

FDA approved FTR based on the findings of phase 3 BRIGHTE clinical trial research for the medical therapy of HIV-1 infection in adult patients with MDR HIV-1 virus infection who are suffering from treatment failure and unable to construct an effective regime [74]. FTR has undergone several clinical trials to ensure its effectiveness and safety before its approval in many research projects.

Treatment with more potent ARV medications, such as FTR, improves HIV-1 patient survival while increasing the risk of complications, such as chronic kidney and liver disease. In the HIV-infected population, liver diseases are becoming more frequent, so in the phase 1 study, researchers evaluated renal and hepatic dysfunction of FTR. In renal (NCT02674581) and hepatic (NCT02467335) clinical trial studies, following a single dose of FTR renal and hepatic dysfunction showed no clinically relevant effect on TMR pharmacokinetics [75]. Also, in an eight-day monotherapy trial study, the antiviral activity, safety, and pharmacokinetics of BMS-488,043, were evaluated. The antiviral activity and safety of this inhibitor were affirmed, but it had weak PK, and its oral bioavailability was limited [76]. In phase 2a (NCT01009814) of the study conducted by Nettles and co-workers focused on the pharmacokinetics, pharmacodynamics, and drug safety of FTR, patients were given varying doses of FTR with or without RTV for eight days. On day eight, the mean reduction in the plasma HIV-1 RNA level at baseline was in the range of 1.21–1.73 log10 copies/mL, and a mean elevation in the CD4 + count from baseline was detected in all regimen groups, with no clinically relevant alterations in the percentages of CD4 and CD8 + T cells. These findings along with the favorable pharmacokinetic profile and usually good tolerability were reported in this clinical trial study [77].

In phase 2b of the study (NCT01384734), 251 patients were placed into five groups through 48 weeks: four in different dosages of FTR and one for atazanavir/ ritonavir (ATV/r) as a control group. During the study, all of these groups received backbone regimes consisting of teltegravir and tenofovir. The FTR groups’ mean CD4 + T-cell count experienced an increase of 145–186 cells/l, while the (ATV/r) group had a mean CD4 + T-cell count increase of 142 cells/l. In this study, FTR doses were safe and there were no FTR-related adverse effects that led to the drug’s withdrawal and indicated similar efficacy compared to (ATV/r) [78]. In another phase 2b study, a randomized, active-controlled trial (NCT01384734), 254 participants enrolled to receive different doses of FTR. The included participants had HIV-1 RNA ≥ 1000 copies/mL and CD4 + cell count > 50 cells/µL and experienced treatment with ARV. Eligible cases were randomized into five groups. All groups received 300 mg of the backbone of Tenofovir disoproxil fumarate (TDF) daily and 400 mg of raltegravir every 12 h. Two cases of each of the first and second groups withdrew consent, and one case of the first group was randomized in error. At week 24, 80%, 69%, 76%, and 72% of treatment groups 1, 2, 3, and 4 obtained an HIV-1 RNA viral load of more than 50 copies/mL, as compared to 75% of the control group [79].

In study conducted by Aberg et al., they have reported 240-week efficacy and safety of FTR + OBT in adults with HIV-1. The results show that 66% of the NRC and 82% of the RC had virologic response. At Week 240, mean change from baseline in CD4 + T-cell count was 296 cells/mm3 (RC) and 240 cells/mm3 (NRC); mean CD4+/CD8 + ratio increased between Weeks 96 and 240. From the Weeks 96 to 240, one additional participant experienced a drug-related serious adverse effect, and six deaths happened. Totally, this study have reported that virologic responses and clinically significant improvements in CD4+/CD8 + ratio and CD4 + T-cell count was observed. The safety and tolerability profile of FTR-based ARV regimens remained consistent with previous studies through 96 weeks, and no new safety trends occurred [80]. In a study conducted by Anderson and co-workers in the 2021 Patient‑Reported Outcomes in phase 3 clinical trial, 371 participants were included in the study and were given at least one dosage of FTR; 272 in the randomized control (RC) (69 placeboes, 203 FTR ) and 99 in the none randomized control (NRC). Through 8 days of functional monotherapy, effectiveness and safety evaluations revealed that FTR (added to the failed regimen) was more effective than placebo. Virologic success (40 copies/ mL, intention-to-treat snapshot analysis) was shown in 53 and 54% of the RC and 37 and 38% of the NRC, respectively, at weeks 24 and 48. FTR was generally well tolerated, with only a small number of adverse events resulting in treatment cessation.

The EQ-5D-3 L, EQ-VAS, and Functional Assessment of HIV Infection (FAHI) tests were used to measure PRO. At week 24, both groups had improved their EQ-5D-3 L US and UK-referenced utility scores from baseline. At week 24, mean visual analog scale (VAS) scores in the RC and NRC rose from baseline by 8.7 (95% CI 6.2–11.2) and 5.6 points (95%CI 1.5–9.7), respectively, and at week 48, they climbed by 9.8 (95% CI 7.0–12.6) and 4.9 points (95% CI 0.6–9.2). From baseline to weeks 24 and 48 in the RC, mean increases in FAHI total score were 6.9 (95% CI 4.2–9.7) and 5.8 (95% CI 2.7–9.0), respectively. Improvements in key EQ-VAS and FAHI domains through week 48, in combination with efficacy and safety data, support the use of FTR for Heavily treatment-experienced people living with HIV-1 [81].

In the BRIGHTE (the name of clinical trial) study, 81% of patients had mild to moderate complications. At week 96, 7% of participants had stopped taking FTR due to an adverse event. Common side effects of the therapeutic dose of FTR (600 mg BID) with optimized backbone therapy include nausea, headache and diarrhea. Most adverse effects were less than 4%, including abdominal pain, pyrexia, dyspepsia, fatigue, asthenia, sleep disorders, vomiting, myalgia, dizziness, pruritus, peripheral neuropathy and immune reconstitution inflammatory syndrome that may occur in patients treated with FTR and backbone therapy [27, 82]. The most important laboratory abnormalities in these patients are related to increases in serum creatinine (greater than 1.8 times the upper limit of normal (ULN)), creatinine kinase (greater than 10 times ULN), bilirubin (greater than 2.6 times ULN), ALT levels (greater than 0.5 times ULN) and AST (Aspartate Aminotransferase) levels (greater than 0.5 times ULN). Other less common laboratory abnormalities like increases in lipase levels (greater than 0.3 times ULN), LDL (low-density lipoprotein) cholesterol levels (greater than 190 mg/dl), triglycerides (greater than 500 mg/dl), urate levels (greater than 12 mg/dl) and blood sugar (greater than 250 mg/dl) may occur. Possible hematological abnormalities such as decreased neutrophil count and hemoglobin levels may also be seen in these patients.

FTR has few adverse reactions at low doses, so it doesn’t require dosage adjustments in patients with renal or hepatic impairment. Immune reconstitution inflammatory is one of the most considerable side effects of using this drug in patients on cART which includes FTR. It may also develop an inflammatory reaction to opportunistic infections like *Mycobacterium avium* infection, *Pneumocystis jirovecii*, cytomegalovirus (CMV), tuberculosis, or pneumonia [82]. Because the use of FTR in patients with hepatitis B or C virus coinfection is linked to an increase in hepatic transaminases, liver function tests should be monitored [27]. At therapeutic levels, FTR has no major negative effects, but doses greater than 2400 mg twice daily can cause QTc prolongation. As a result, FTR should be used with caution in individuals on other medications that can prolong the QT interval [73].

Some medicines like Ethinyl estradiol and statins, when taken together with FTR may increase their concentration and induce side effects [61, 83]. There are inadequate human data on the use of RUKOBIA during pregnancy to accurately estimate the risk of birth abnormalities and miscarriage related with the medicine. During organogenesis, oral administration of FTR to pregnant rats and rabbits had no adverse developmental effects at clinically relevant TMR exposures in animal reproduction experiments. However, research is still ongoing to ensure that the FTR is safe during pregnancy.

Information on using FTR during pregnancy is not sufficient to precisely evaluate the risk of birth defects and miscarriage related to drugs. It’s unclear whether FTR is found in human breast milk, whether it affects human milk production, or whether it affects the breastfed child. A FTR-related medication was found in rat milk after it was given to nursing rats. Therefore, the use of this drug during breastfeeding should be done with caution. FTR is not approved for children use and in pediatric patients; the safety and efficacy of FTR have not been demonstrated. The number of patients aged 65 and older in FTR clinical studies was insufficient to assess if they respond differently than younger ones. In general, while administering FTR to older patients with reduced hepatic, renal, or cardiac function, as well as concurrent illness or other pharmacological therapy, caution should be given [82, 84].

## Conclusion and outlook

For more than a decade, therapeutic HIV recommendations have included ART, which has saved the lives of countless HIV/AIDS patients. Extraordinary increased usage of HIV medications has coincided with the establishment of HIV drug resistance. Due to the advent of drug-resistant virus, all ARV medications, including those from newer pharmacological classes, are in danger of becoming partially or completely inactive. HIV medication resistance, if not controlled might impair the efficacy of HIV treatments leading to an increase in HIV infections. FTR is currently in an ongoing phase 4 and post-marketing studies. Also, many studies on the effectiveness and side effects of this drug in children, elderly patients, pregnant and lactating women are under investigation. However, after passing 3 phases of clinical trial studies, this drug has the appropriate effectiveness with minimal side effects and interactions. Heavily treatment-experienced adults with MDR HIV-1 have many problems, including using multiple drugs with low efficacy. They also experience high side effects and high drug interactions due to polypharmacy. Therefore, it seems logical that considering the risk/benefit ratio, FTR can be a suitable alternative treatment for these patients.

## Data Availability

All the data in this review are included in the manuscript.
